# Changes in skinfold thickness and waist circumference after 12 and 24 months resulting from the NHF-NRG In Balance-project

**DOI:** 10.1186/1479-5868-7-26

**Published:** 2010-04-07

**Authors:** Lydia Kwak, Stef PJ Kremers, Math JJM Candel, Tommy LS Visscher, Johannes Brug, Marleen A van Baak

**Affiliations:** 1 Unit for Preventive Nutrition, Department of Biosciences, Karolinska Institutet, Huddinge, Sweden; 2 Department of Health Promotion, Maastricht University, Maastricht, the Netherlands; 3 Department of Methodology and Statistics, Maastricht University, Maastricht, the Netherlands; 4 Institute of Health Sciences, Vrije Universiteit, Amsterdam, the Netherlands; 5 EMGO Institute, VU University Medical Centre, Amsterdam, the Netherlands; 6 Department of Human Biology, Maastricht University, Maastricht, the Netherlands

## Abstract

**Background:**

More knowledge is needed regarding the effectiveness of weight gain prevention programmes. The present study tested the 12-and 24-month effectiveness of the 'Netherlands Research programme weight Gain prevention' (NHF-NRG)-In Balance-project, a worksite-based intervention aimed at the prevention of weight gain.

**Methods:**

Twelve worksites (n = 553 participants) were matched and assigned to either intervention or control group. The worksites and employees of the intervention group received individual (i.e. pedometer, computer-tailored advice) and environmental (i.e. changes in worksite canteen) interventions, directed at physical activity and food intake over 1-year. Differences between the intervention and control group in changes in body weight, BMI, skinfold thickness and waist circumference at 12 and 24 months were examined using multilevel linear regression analyses adjusting for various baseline characteristics (age, gender, BMI, marital status, education and smoking status).

**Results:**

A significant greater reduction in skinfold thickness was found in the intervention group than in the control group, both after 12-and 24 months (Unstandardized regression coefficients (B) = -2.52, 95% C.I. -4.58, -0.45; p = 0.018; B = -4.83, 95% C.I. 6.98, -2.67; p < 0.001 respectively). Significant differences were also observed for changes in waist circumferences both at 12 months (B = -1.50, 95% C.I. -2.35, -0.65; p < 0.001) and at 24 months (B = -1.30, 95% C.I. -2.18, -0.42; p = 0.005). No significant changes were observed for weight and BMI.

**Conclusions:**

The project was effective with regard to changes in skinfold thickness and waist circumference both at 12 and 24 months. It supports the usefulness of worksite-based prevention, especially regarding maintenance of behavioral changes.

## Background

Data from around the world show alarming increases in the prevalence of obesity during past decades [1]. The situation in the Netherlands is no exception, with comparable increases in the number of individuals suffering from overweight and obesity as other European countries. In order to tackle this health issue it has been suggested that efforts should focus on promoting small life-style changes and not on producing weight loss, but on eliminating or reducing the gradual excessive weight gain occurring in people of all ages [2]. However, despite the expanding interest, little is known about the effectiveness of weight gain prevention programmes, as few studies have tested programmes that were designed for this purpose [3,4].

The multidisciplinary research programme 'Netherlands Research programme weight Gain prevention' (NHF-NRG) was initiated for exactly this purpose and aimed to evaluate three weight gain prevention programmes targeted at different risk-groups [5]. The present paper describes the effectiveness of one of these programmes, namely the NHF-NRG In Balance-project: a worksite-based prevention programme directed at the prevention of weight gain in young adults, through changes in both physical activity and food intake [6]. The programme, which was developed based on the Intervention Mapping protocol [7], aimed to prevent weight gain through the following programme objectives: (1) increase frequency and duration of walking and cycling, (2) increase physical activity level at work, (3) decrease portion sizes and (4) decrease intake of energy-dense foods, through replacement of high fat products by low energy dense products, replacement of products low in fibre by fibre-rich products and replacement of saturated fats by unsaturated fats.

Young adults are of particular relevance for prevention of weight gain, as young adulthood is recognized as a high-risk period for weight gain [8,9]. The average annual weight gain of young adults in the Netherlands is approximately 0.60 kg/year [10]. The NHF-NRG In Balance-project was worksite-based, not only as this is a major environmental context for young adults [11], but also because worksites provide many opportunities to reinforce the adoption and maintenance of healthy lifestyle behaviours [5] Moreover, worksites provide the opportunity to deliver interventions across multiple levels of influence, including individual, interpersonal and environmental influences [12]. Previous worksite health promotion programs that have combined interventions both for individuals and workplace environment, such as the Seattle 5-a-Day worksite study [13], the Working Healthy Project [14] and the WellWorks-2 study [15], have been found to be effective. In the NHF-NRG In Balance-project a systems-approach to the intervention was therefore applied, combing individual interventions with environmental interventions designed to support the healthy behaviours.

The present paper reports the 12-and 24-month results of the NHF-NRG In Balance-project on several anthropometric measures, which include body weight, body mass index (BMI), skinfold thickness and waist circumference.

## Methods

### Participants

**Figure 1:**
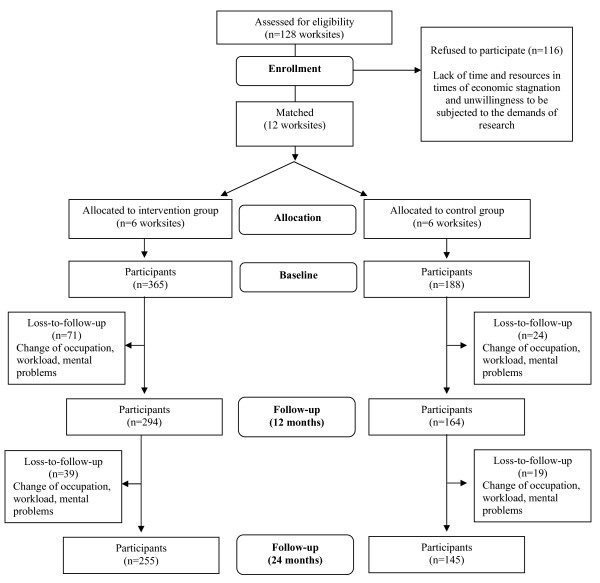
**Flowchart NHF-NRG In Balance-project**.

The recruitment of participants included two steps. First, worksites were recruited based on data collected through the Chamber of Commerce. Eligible worksites should have a minimum of 100 employees and provide canteen facilities. In total, a random selection of 128 worksites located in the south of the Netherlands and meeting the inclusion criteria, was approached by mail and telephone and invited to participate. Interested companies were informed of the project and research demands. During the recruitment, it became apparent that worksites were unwilling to participate due to the randomization design of the study; six worksites (a hospital, local government, paper factory, tile factory, pigment factory and water-supplying company) agreed to participate in the study however only if they were part of the intervention group [16]. Consequently, the randomization design was dropped and a quasi-experimental pre-test multiple-post-test control group design was applied. Accordingly, six control worksites (a hospital, local government, furniture manufacturer, cable factory, energy-supplying company and a university) were matched with respect to the social economic status of the worksites of the intervention group. These differences in social economic status of the worksites were assessed by the occupational level of the worksite, blue versus white collar employees (see also [16]). The participating worksites included all the departments within these worksites and employed 100-800 persons (see figure 1). The overall participation rate of worksites was 9%.

The second part of the recruitment concerned the individual participants (employees). With permission of the participating worksites, all employees under 40 years of age were contacted by letter and invited to participate in a weight gain prevention programme on a voluntary basis. Only participants with a BMI above 18 kg/m^2 ^were eligible for the study. Additionally, participants were excluded if they had any medical restrictions with regard to diet or physical activity behaviour. Interested individuals (20-25% per worksite) received an information booklet in which the project was described in more detail and which included an informed consent form. Those who showed an interest were all included in the study. Both participants of the intervention and control group read and signed the same informed consent form before participating in the study. The study was approved by the Medical Ethical Committee of the Academic Hospital Maastricht, the Netherlands.

### The NHF-NRG In Balance-project intervention

**Figure 2:**
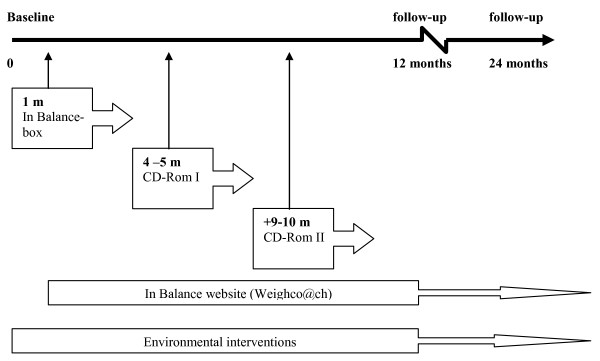
**Three stage intervention sequence**.

A complete description of the intervention of the NHF-NRG In Balance-project has been published previously [6]. Briefly, the programme consisted of an individual component and a worksite (environmental) component; both were directed at changes in food intake and physical activity (see figure 2).

Participants in the intervention group received all the interventions of the individual component of the NHF-NRG In Balance-programme. However, the worksites of the intervention group were given the freedom to implement environmental interventions, which the linkage-boards found suitable. Linkage-boards are systems that connect those who are developing the programme to those who will be using the programme, in order to encourage collaborative program development ending in effective implementation [7]. Worksites and individuals in the control group did not receive any interventions and were contacted only for measurements. Participants were told that they were participating in a study aimed at monitoring body composition changes over a period of two years. After the study-period, they received certain components of the NHF-NRG In Balance-project.

### Individual interventions

The individual component contained the following interventions: expert monitoring and evaluation of body composition measures in relation to healthy standards; 'In Balance-box' consisting of a pedometer, waist circumference measuring tape (indicating with a colour-scheme if one has a healthy waist circumference), a 'calorie-guide' and instruction brochure (including physical activity and food intake diaries, log of steps walked); In Balance-website (includes access to WeightCo@ch, a personalised advice instrument aimed at weight maintenance); two computer-tailored CD-ROMs. Both CD-ROMs gave stage-matched tailored feedback; the first was directed at awareness of weight status and knowledge regarding the energy balance-related behaviours, the second at changing the energy balance-related behaviours. We tailored on participant's choice of behavioural priority of change [17] by giving the participants the choice which energy balance-related behaviour they would like to change first.

### Environmental interventions

The environmental components were to be delivered by a worksite linkage board [7] within each worksite over a continuous period. The linkage-board comprised of a representative of the research team (only the first year, see figure 2) and employees who were able to facilitate collaboration or were in the positions to influence changes necessary to support the adoption, implementation and institutionalisation of the programme, such as representatives of the worksite cafeteria, of human resources, care-taker, dietician etc. Environmental interventions were incorporated into a handbook, which contained the existing 7-step programme of the Workplace Health Promotion consultancy (The Netherlands Institute for Health Promotion and Disease Prevention (Healthcare and Work)) and served as a guide for assisting the worksite linkage board through the different stages of diffusion. The board was free to choose which environmental intervention they wanted to implement within their company, if necessary environmental interventions could be modified to better fit within the specific worksite. The selection process of suitable environmental interventions was dependent on the needs and possibilities of the worksite. Interventions included for example changes in the assortment of food products in the cafeteria, workshops, an information wall containing information on the balance between food intake and physical activity, posters/prompts stimulating stair use and ways to form lunch-walking and cycling groups.

### Outcome measures

Outcome measures were assessed at baseline, 12 and 24 months and included body composition measures and self-report measures. The protocol for the measurements was identical for all three time-points and for all subjects. All measurements were executed in the morning after an overnight fast, at the worksite of the participant and performed by the same researcher. Measurements started in September 2003 and data collection was completed for all study participants in August 2006.

Body weight, height, skinfold thickness and waist circumference were measured. Body weight (kg) was measured, in underwear, to the nearest 0.1 kg with a digital laboratory scale (Seca, Model 861, Hamburg, Germany). Height (m) was measured to the nearest 1.0 mm without shoes with a mobile measuring unit (Seca, Model 225, Hamburg, Germany). Skinfold thickness was determined using the sum of four skinfolds measured with the Harpenden skinfold calliper (HSK-BI, British Indicators, West Sussex, UK). Skinfolds included biceps (anterior surface of the biceps midway between the anterior auxiliary fold and the antecubital fossa), triceps (vertical fold on the posterior midline of the upper arm, halfway between the acromion and olecranon process), subscapular (fold on the diagonal line coming from the vertebral border to between 1 and 2 cm from the inferior angle of the scapulae) and suprailiac (diagonal fold above the iliac crest even with the anterior auxiliary line). Waist circumference measures were obtained to the nearest 0.1 cm with a tape measure. It was measured at the abdominal waist (horizontal at the umbilicus).

The self-report measures were assessed with a self-administered written questionnaire, which participants returned completed during the body composition measurements. The following self-report outcome measures obtained by the questionnaire were incorporated in the present study: demographic characteristics including gender, date of birth, marital status (married or living together; separated, divorced, widowed, never married) and highest level of education (high school or less; some college/vocational training; university); smoking status, measured with the following item: "do you smoke?" (yes, daily; yes, occasionally; no, never).

### Statistical analyses

A required sample size of 500 participants from 12 worksites was determined to be large enough to detect a medium effect size (Cohen's *d *0.45), with a power of 80% and an alpha of 0.025, which is corrected for multiple testing. The power calculation assumed an intraclass correlation of 0.02, which results in a required sample size of 465, taking a 20% drop-out rate into consideration. The primary outcomes examined in this study are changes in body weight, BMI, sum of skinfolds and waist circumference from baseline to 12 months and 24 months. Prior to the analyses, data of female participants who had been pregnant during the two-year project were excluded. To assess potential dropout bias, baseline characteristics age, gender, BMI, marital status, education and smoking status were compared between those who dropped-out and those who attended all measurements. An intention-to-treat analysis was conducted for 12 and 24 months in which dropouts in the intervention and control group were assigned average weight changes that were observed in the control group at both time-points. The analyses (data not presented) showed similar results as the 'on treatment analyses' both for the changes after 12 and 24 months, the 'on treatment analysis' are the primary analyses and are presented below.

The effect analyses were performed in five steps. First, differences in baseline characteristics of participants in the intervention and control group were explored, using Student's t-test or Chi-square test. Second, differences between the intervention and control group in changes in body weight, BMI, skinfold thickness and waist circumference at 12 and 24 months were examined using linear regression analyses adjusting for various baseline characteristics (age, gender, BMI, marital status, education and smoking status). The measurements were repeatedly obtained for the same subjects, nested within several worksites, yielding a three level design. To deal with possible dependencies in the measurements across time due to being obtained for the same worksites and persons, the multilevel linear regression analyses were conducted in MlwiN employing a random intercept that varies both at the level of worksites and at the level of persons [18]. By including the baseline measurement of the outcome variable in the analysis as one of the measurements at the lowest level, combined with a specific coding for the effect of time, differences of outcomes with baseline are analysed, in this way correcting for differences between the intervention groups at baseline. In this analysis the unstandardized regression coefficient (B) for the interaction between time and the intervention factor represents the intervention effect on such change scores. The analysis model is comparable to a repeated measures ANOVA (adjusted for baseline) for follow-ups at 12 months and at 24 months, however there being no random interaction effects with time. Adjustments for the baseline value of age, gender, BMI, marital status, education and smoking status were made, by including these variables as covariates in the analysis. All p-values are two-sided and 5% level of significance was used. Thirdly, Cohen's *d *effect sizes were calculated in order to calculate the magnitude of the intervention effect; *d *is defined as the difference between two means divided by the pooled standard deviation in the population. Effect sizes are defined as "small, *d *= .2," "medium, *d *= .5," and "large, *d *= .8" [19]. Fourth, potential interaction effects of the intervention group with gender, age and BMI were explored. If significant interactions occurred analyses were repeated with stratification by gender, age, or BMI. Fifth, two types of intraclass correlation coefficients (ICCs) were calculated for each of the four outcome measures. The ICC on worksite-level is the random intercept variance at worksite-level divided by the total variance and thus reflects the degree to which differences on outcome measures can be explained by random effects of the worksites. The ICC on person level is the random intercept variance at worksite level plus the random intercept variance at person level divided by the total variance, thus reflecting to what extent differences on outcome measures can be explained by random effects of worksites and individuals [20].

## Results

### Attendance

The number of participants who were not measured at 12 and 24 months was 71 (19.5%) and 110 (30.1%), respectively, for the intervention group and 24 (12.8%) and 43 (22.9%), respectively, for the control group (see flowchart). The most common reasons for discontinuation were change of occupation, conflict with workload and stress-related issues. The dropout analyses revealed some selective dropout. Both after 12 and 24 months smokers were more likely to discontinue the study than non-smokers (OR = 1.87, 95% C.I. 1.02-3.43 and OR = 2.33, 95% C.I. 1.41-3.84).

### Participant characteristics

Baseline characteristics of the control and intervention group are described in table 1. Participants from the intervention group were older and had a higher BMI than participants from the control group (38.9 vs. 35 years and 25.7 vs. 24.2 kg/m^2^). Apart from these differences groups did not differ in terms of baseline characteristics. Overall, there was an equal distribution of men and women between the intervention and control group and participants were equally well educated, nearly 50% had tertiary education, married or living together and non-smokers.

**Table 1:** Baseline characteristics of participants

	Control (n = 188)	Intervention (n = 365)	P-value
Age, years mean (s.d.)	35.0 (7.4)	38.9 (8.2)	<.01
Gender (% female)	48.2	50.7	.61
Highest education (%)			.41
High school or less	19.3	15.7	
Some college/vocational training	29.2	34.7	
University	51.6	49.6	
Marital status (%)			.06
Married or living together	77.1	83.9	
Separated, divorced, widowed or never married	22.9	16.1	
Current smoker (%)	17.3	16.0	.72
Body Mass Index, kg/m^2 ^mean (s.d.)	24.2 (3.1)	25.7 (4.0)	<.01

*Note: *Abbreviation: s.d., standard deviation. P-value estimated by t-test, with exception for education and marital status, which was estimated with Chi-square tests.

### Anthropometric changes over 12 and 24 months

Changes in skinfold thickness, waist circumference, body weight and BMI for the two groups over 12 and 24 months are depicted in table S1 (additional file 1). At 12 months follow-up, a statistically significant difference between the intervention and control group was evident for the change of sum of skinfolds (B = -2.52, 95% C.I. -4.58, -0.45), with a corresponding Cohen's *d *of 0.26. A greater reduction in sum of skinfolds was observed for participants in the intervention group than for participants in the control group. These differences sustained over 24 months (B = -4.83, 95% C.I. -6.98, -2.67), the Cohen's *d *was 0.44. Significant differences were also observed between the intervention and control group for changes in waist circumferences both at 12 months (B = -1.50, 95% C.I. -2.35, -0.65) and at 24 months (B = -1.30, 95% C.I. -2.18, -0.42), with respectively Cohen's *d *0.37 and 0.33. Participants from the intervention group reduced their waist circumferences over time in comparison to an increase in the control group.

Changes in weight and BMI however did not differ significantly between the two groups neither at 12 nor at 24 months. Although changes in weight and BMI were not statistically significant, they were in favour of the intervention group. The corresponding Cohen's *d*'s were all smaller than 0.20.

Significant interaction terms were found for the changes in skinfold thickness (table 2). A significant interaction effect of group with gender was observed for the changes in skinfold thickness at 12 (p < .05) and 24 months (p < .05), on the basis of which a stratification was made by gender. A larger reduction in skinfold thickness was observed in women in the intervention group than in women in the control group both at 12 months (B = -3.15, 95% C.I. -5.95, -0.35) and at 24 months (B = -5.14, 95% C.I. -8.06, -2.21). No significant effects were observed among men.

**Table 2:** Body composition changes stratified for gender adjusted for baseline age, BMI, marital status, education and smoking status

	Men				Women			
	**I**	**C**	**B** **95% C.I**.	**P-value**	**I**	**C**	**B** **95% C.I**.	**P-value**

Skinfolds thickness (mm)								
12 m	-6.0 ± 10.8 (N = 144)	-5.4 ± 7.5 (N = 86)	1.27 -1.60, 4.13	.388	-8.6 ± 12.0 (N = 150)	-3.8 ± 10.5 (N = 78)	-3.15 -5.95, -0.35	.030
24 m	-7.4 ± 12.0 (N = 128)	-5.3 ± 10.1 (N = 76)	0.06 -2.96, 3.08	.970	-12.6 ± 12.8 (N = 127)	-4.7 ± 10.7 (N = 69)	-5.14 -8.06, -2.21	<.001

*Note: *Abbreviations: intervention group (I); control group (C); unstandardized regression coefficient (B); sample size (N), months (m). P-values for differences between the intervention and control group.

### Process evaluation of the environmental interventions

Data collected by observation and registration of activities revealed that four of the six worksites implemented environmental interventions. All four worksites placed posters near the elevators and stairs to stimulate stair use over a 3-week period [21] and provided general information on the project. Two (hospital, paper-factory) of these four worksites formed worksite linkage boards and implemented more environmental interventions, which included making the NHF-NRG In Balance-project visible through articles in the worksite personnel magazine or through intranet. The hospital organized several special events: a 1-week placement of an 'information wall' containing information on the balance between food intake and physical activity in addition to the presence of a health professional who took waist circumference measurements and gave advice. This worksite also handed out free apples during National Health Week, together with information booklets and maps and walking routes that were located around the hospital. Moreover, they made their personnel aware of the hospitals physical activity facilities, e.g. squash, aerobic classes, bikes to borrow. After the 2-year period the hospital was in negotiation regarding a specific bike-scheme. The paper-factory organized a series of workshops given by a dietician on healthy eating, distributed pamphlets on physical activity and information regarding special offers at local sports facilities.

## Discussion

The present study was designed to test the 12-and 24-month effectiveness of the NHF-NRG In Balance-project, with regard to changes in body weight, BMI, sum of skinfolds and waist circumference. The results indicate that with regard to changes in sum of skinfolds and waist circumference the project was indeed effective at both 12 and 24 months. Even though changes in weight and BMI between the intervention and control group were not significantly different, they did change in the desired direction. Overall, the intervention of the NHF-NRG In Balance-project had a positive effect on the body composition measures of the individuals in the intervention group. The interpretation of effect sizes of Cohen's *d *imply effects of medium magnitude for the changes in skinfold thickness and waist circumference both after 12 and 24 months (Cohen's *d *between 0.33 and 0.55). Such changes in body composition indicators may have important health implications, as it has been demonstrated that the health risks associated with obesity derive primarily from fat rather than weight [22]. Moreover, it is not only the total amount of fat that is important, but also the distribution of fat in the body [23], with central fatness being most related to health risks [24]. The reduction in skinfold thickness and waist circumference observed in the present study reflects a reduction in central fatness [22,25]. The decrease in waist circumference is most relevant, as a large waist circumference is independently associated with health risks [26,27] and mortality [28,29]. On a population level it has even been shown that there is a more significant trend of increases in waist circumference over time than BMI [30].

The observed changes in anthropometric measures could be a result of changes in participants' food intake and/or physical activity behaviour. With regard to changes in waist circumference it has been demonstrated that an increase in fibre intake was associated with a reduction in waist circumference in men [31]. A strong dose-response relationship has also been observed between the amount of exercise and measures of central obesity [32]. Interestingly, changes in physical activity can lead to changes in body composition, which may be reflected in changes in waist circumference, while body weight remains stable through increased muscle mass [33,34]. This is in line with the findings of the present study.

Stratified outcome analyses were interesting. It appeared that the intervention only had an effect on the changes in skinfold thickness in women and not in men. It would be interesting to see if this is a result of the engagement in different energy balance-related behaviours of men and women.

The process evaluation of the environmental interventions showed that two worksites formed a worksite linkage-board, who implemented several environmental interventions throughout the two year period. When taking baseline characterises into consideration, the individuals in these two worksites appeared to show better results with regard to changes in waist circumference and sum of skinfolds than individuals in worksites with fewer components to the intervention both after 12 and 24 months (data not shown). Although the study was not powered to significantly detect these between-worksite differences, this finding does underscore the importance of intervening on both the individual and the environmental level. Moreover, it showed that the context of the worksites did not affect the uptake of the intervention, as one of these two worksites had predominantly white-collar workers and the other blue-collar. This finding as worksite-health promotion programs are often less likely to result in health behaviour change in blue-collar workers [35].

The NHF-NRG In Balance-project is one of few worksite obesity prevention programmes, which 1) is primarily aimed at weight gain prevention through changes in both food intake and physical activity, 2) contains both individual and environmental components and 3) assesses longer-term follow-up effectiveness. A recent review of papers on lifestyle interventions aimed at prevention of overweight and obesity, with primary programme objective weight management, prevention of weight gain or moderate weight loss among adults, included four additional studies to the present study, in which workplace interventions were evaluated. Two of these studies included behavioural goals that were aimed at both diet and physical activity; three included both cognitive and environmental goals and two studies assessed effectiveness after a 12 month follow-up. Significantly smaller increases in BMI in the intervention conditions were observed in one study; no treatment effect for weight or BMI changes was found in the others. Two of the studies also included measurements on percent body fat, both of which observed significantly positive effects [36]. These findings are in line with those observed in the present study. To date, there has been an increase in the number of worksite obesity prevention studies that are testing environmental or combined environmental-and individual-level worksite interventions over a longer period of time, e.g., through the National Heart, Lung, and Blood Institute [37]. However results regarding effectiveness have not yet been published.

In the present study, we perceived several benefits of implementing the intervention within a worksite setting. Firstly, the worksites provided access to a large number of adults with different educational backgrounds. Moreover, the employees within the worksites are able to play an important role in diffusing the intervention throughout the worksite by impacting social norms, which in the long-term may influence the behaviours of co-workers who did not change their behaviour initially [38]. Difficulties were perceived with regard to enhancing facilitators of environmental changes, as only two of the six worksites set up a worksite-linkage board. As the linkage boards play a crucial role in the adoption, implementation and institutionalization of the environmental components, strategies should be developed to mobilize support and commitment for the formation of such boards.

There are a number of limitations of this study, including those concerning the generalizability. The first is related to the recruitment of companies, as only 9% of the approached companies were willing to participate. An important reason for companies not to participate in the NHF-NRG In Balance-project proved to be the randomized evaluation design of the programme, implying that companies were not willing to take the risk of being excluded from the intervention [16]. We were therefore forced to drop the original randomization design of the programme and assign worksites to the experimental and control group based on matching. As a result of which it is possible that selection bias occurred, weakening the internal validity of the results. Moreover, external validity was weakened by the fact that participating worksites were most likely not representative of the average worksite, in that the participating worksites probably showed a higher interest in health promotion than worksites in general. Implementing the project in less interested worksites might not have generated the same results. A second limitation of the present study is the recruitment of participants. Even though the aim of the project was to prevent weight gain in young adults, there was a relatively high response of older and overweight individuals, in line with observations of other studies [27,28]. This may have resulted in a selection bias, in which individuals who were more interested to change the targeted behaviours were oversampled. Moreover, there was a high response of participants with a tertiary education. The third limitation concerns the statistical analysis, although sophisticated multilevel analyses were executed in this study, the statistical procedures may not fully account for all potential dependencies that were introduced as a result of the research design. For example, our statistical model contained only one random component for worksite, implying that every worksite is assumed to have exactly the same response to the intervention (if in intervention) or to the control situation (if in the control condition). The fourth limitation pertains to the process evaluation; unfortunately we were unable to perform an in-depth analysis regarding the uptake of interventions by the individuals. The fifth limitation is related to the absence of a significant difference in weight changes over time between both groups. Even though the drop-out rate after two years was above 20% (27.5%), the required sample size to detect a medium effect (n = 372) was still met (n = 401). However, weight changes observed in the control group were smaller than those expected, with smaller weight change differences between the groups (0.5 kg at 12 months). The smaller increase in weight in the control group is most likely a result of measurement effects. However, it could also be a result of a selection bias; the control group might have consisted of more motivated individuals who are susceptible to change. Moreover, it is possible that those individuals who dropped-out were those with a higher BMI.

## Conclusions

The findings presented here show the effectiveness of the NHF-NRG In Balance-project and support the value of using workplace settings for maintenance of behavioural changes in the area of weight gain prevention. Additionally, it underscores the importance of systematically developing an intervention that contains both individual and environmental components and is directed at changing both physical activity and dietary behaviour. Furthermore, the results support the notion that more attention needs to be given to generating interest in weight management both among worksites and among individuals who are at risk of weight gain.

## Competing interests

The authors declare that they have no competing interests.

## Authors' contributions

LK conducted the study, analyzed the data and conceived and drafted the original manuscript. SPJK and MJJMC assisted with the statistical analyses. SPJK, TLSV, JB and MvB provided critical feedback on drafts. All authors read and approved the final manuscript.

## Supplementary Material

Additional file 1 **Table S1 - Estimates of treatment effect (B), intercept variance and the intra-class correlation coefficients**. *Note: *Random intercept at worksite and person level, adjusted for baseline age, gender, BMI, education, marital status and smoking status. P-values for differences between intervention and control groups. The ICC (worksite) is the random intercept variance at worksite level divided by the total variance of the outcome measure, the ICC (person) is the random intercept variance at worksite level plus the random intercept variance at person level divided by the total variance of the outcome measure. Abbreviations: sample size (N); standard deviation (SD); unstandardized regression coefficient (B), Confidence Intervals (C.I), estimates of random intercept variance (s^2^), Standard Error (SE), Intraclass Coefficient (ICC). Click here for file

## References

[B1] World Health Organization World Health Organization Consultation on Obesity Obesity: preventing and managing the global epidemic (WHO technical report series, 894). Geneva 2000 11234459

[B2] Kemper HCG Stasse-Wolthuis M Bosman W The prevention and treatment of overweight and obesity: summary of the advisory report by the Health Council of the Netherlands Neth J Med 2004 62 10 17 15061227

[B3] Hill JO Can a small-change approach help address the obesity epidemic? A report of the Joint Task Force of the American Society for Nutrition, Institute of Food Technologists, and International Food Information Council Am J Clin Nutr 2009 89 477 484 10.3945/ajcn.2008.26566 19088151

[B4] Hardeman W Griffin S Johnston M Kinmonth AL Wareham NJ Interventions to prevent weight gain: a systematic review of psychological models and behaviour change methods Int J Obes 2000 24 131 143 10.1038/sj.ijo.0801100 10702762

[B5] Kremers SPJ Visscher TL Brug J Chin A Paw MJ Schouten EG Schuit AJ Netherlands research programme weight gain prevention (NHF-NRG): rationale, objectives and strategies Eur J Clin Nutr 2005 59 498 507 10.1038/sj.ejcn.1602100 15714217

[B6] Kwak L Kremers SPJ Werkman A Visscher TLS Van Baak MA Brug J The NHF-NRG In Balance-project: the application of Intervention Mapping in the development, implementation and evaluation of weight gain prevention at the worksite Obes Rev 2007 8 347 61 10.1111/j.1467-789X.2006.00304.x 17578384

[B7] Bartholomew LK Parcel GS Kok G Gottlieb NH Intervention Mapping: Designing theory-and evidence-based health promotion programs. Mountain View, Mayfield 2001

[B8] Sherwood NE Jeffery RW French SA Hannan PJ Murray DM Predictors of weight gain in the Pound of Prevention study Int J Obes 2000 24 395 403 10.1038/sj.ijo.0801169 10805494

[B9] Sheehan TJ DuBrava S DeChello LM Fang Z Rates of weight change for black and white Americans over a twenty year period Int J Obes 2003 27 498 504 10.1038/sj.ijo.0802263 12664083

[B10] Visscher TLS Kromhout D Seidell JC Long-term and recent time trends in the prevalence of obesity among Dutch men and women Int J Obes 2002 26 1218 1224 10.1038/sj.ijo.0802016 12187399

[B11] Dishman RK Oldenburg B O'Neal H Shephard RJ Worksite physical activity interventions Am J Prev Med 1998 15 344 361 10.1016/S0749-3797(98)00077-4 9838977

[B12] Sorensen G Stoddard A Hunt MK Hebert JR Ockene JK Spitz Avrunin J The effects of a health promotion-health protection intervention on behaviour change: the WellWorks Study Am J Public Health 1998 88 1685 1690 10.2105/AJPH.88.11.1685 9807537 PMC1508574

[B13] Beresford SAA Thompson B Feng Z Christianson A McLerran D Patrick DL Seattle 5-A-Day worksite program to increase fruit and vegetable consumption Prev Med 2001 32 230 238 10.1006/pmed.2000.0806 11277680

[B14] Emmons K Linnan LA Shadel WG Marcus B Abrams DB The Working Healthy Project: a worksite health promotion trial targeting physical activity, diet and smoking J Occup Environ Med 1999 41 7 545 555 10.1097/00043764-199907000-00003 10412096

[B15] Sorenson G Stoddard AM LaMontagne AD Emmons K Hunt MK Youngstrom R A comprehensive worksite cancer prevention intervention: behaviour change results from a randomized controlled trial (United States) Cancer Causes and Control 2002 13 493 502 10.1023/A:1016385001695 12195637

[B16] Kwak L Kremers SPJ Van Baak MA Brug J Participation rates in worksite-based intervention studies: health promotion context as a crucial quality criterion Health Promot Int 2006 21 66 69 10.1093/heapro/dai033 16339773

[B17] Campbell MK Tessaro I DeVellis B Benedict S Kelsey K Belton L Tailoring and targeting a worksite health promotion program to address multiple health behaviours among blue-collar women Am J Health Prom 2000 14 306 313 10.4278/0890-1171-14.5.306 11009857

[B18] Rabash J Browne W Goldstein H Yang M Plewis I Draper D Healy M Woodhoose G A user's guide to MlwiN 1999 London, Institute of Education

[B19] Cohen J Statistical power analysis for the behavioural sciences 1998 Hillsdale NJ, Lawrence Earlbaum Associate

[B20] Murray DM The design and analysis of group randomized trials 1998 NY: Oxford University Press

[B21] Kwak L Kremers SPJ van Baak M Brug J A poster-based intervention to promote stair use in blue-and white-collar worksites Prev Med 2007 45 177 181 10.1016/j.ypmed.2007.05.005 17610944

[B22] Wells JCK Victoria CG Indices of whole-body and central adiposity for evaluating the metabolic load of obesity Int J Obes 2005 29 483 489 10.1038/sj.ijo.0803054 15672103

[B23] Pi-Sunyer FX Obesity: criteria and classification Proc Nutr Soc 2000 59 505 509 11115784 10.1017/s0029665100000732

[B24] Seidell JC Deurenberg P Hautvast JG Obesity and fat distribution in relation to health-current insights and recommendations World Rev Nutr Diet 1987 50 57 91 3300052 10.1159/000414170

[B25] Lemieux S Prud'homme D Bouchard C Tremblay A Després J A single threshold value of waist girth identifies normal-weight and overweight subjects with excess visceral adipose tissue Am J Clin Nutr 1996 64 685 693 8901786 10.1093/ajcn/64.5.685

[B26] Chan JM Rimm EB Colditz GA Stampfer MJ Willet WC Obesity, fat distribution, and weight gain risk as risk factors for clinical diabetes in men Diabetes Care 1994 17 961 969 10.2337/diacare.17.9.961 7988316

[B27] Rimm EB Stampfer MJ Giovannucci E Ascherio A Spiegelman D Colditz GA Willett WC Body size and fat distribution as predictors of coronary heart disease among middle-aged and older US men Am J Epidemiol 1995 148 1187 1194 10.1093/oxfordjournals.aje.a117385 7771450

[B28] Baik I Ascherio A Rimm EB Giovannucci E Spiegelman D Stampfer MJ Willett WC Adiposity and mortality in men Am J Epidemiol 2000 152 264 271 10.1093/aje/152.3.264 10933273

[B29] Visscher TLS Seidell J Molarius A Kuip D Van der Hofman A Witteman J A comparison of body mass index, waist-hip ratio and waist circumference as predictors of all-cause mortality among the elderly: the Rotterdam study Int J Obes 2001 25 1730 1735 10.1038/sj.ijo.0801787 11753597

[B30] Chen R Tunstall-Pedoe H Socioeconomic deprivation and waist circumference in men and women: the Scottish MONICA surveys 1989-1995 Eur J Epidemiol 2005 20 141 147 10.1007/s10654-004-4498-y 15792280

[B31] Koh-Banerjee P Chu NF Spiegelman D Rosner B Colditz G Willet W Rimm E Prospective study of the association of changes in dietary intake, physical activity, alcohol consumption, and smoking with 9-y gain in waist circumference among 16 587 US men Am J Clin Nutr 2003 78 719 727 14522729 10.1093/ajcn/78.4.719

[B32] Slentz CA Duscha BD Johnson JL Ketchum K Aiken LB Samsa GP Effects of the amount of exercise on body weight, body composition, and measures of central obesity: STRRIDE a randomized controlled study Arch Intern Med 2004 164 31 39 10.1001/archinte.164.1.31 14718319

[B33] Wilmore JH Després JP Stanforth PR Mandel S Rice T Gagnon J Alterations in body weight and composition consequent to 20 wk of endurance training: the HERITAGE family study Am J Clin Nutr 1999 70 346 352 10479196 10.1093/ajcn/70.3.346

[B34] Ross R Dagnone D Jones PJ Smith H Paddags A Hudson R Janssen I Reduction in obesity and related comorbid conditions after diet-induced weight loss or exercise-induced weight loss in men. A randomized, controlled trial Ann Intern Med 2000 133 92 103 10896648 10.7326/0003-4819-133-2-200007180-00008

[B35] Niknian M Linnan L Lasater T Carleton R Use of population-based data to assess risk factor profiles of blue and white collar workers J Occup Med 1991 33 29 36 10.1097/00043764-199101000-00010 1995799

[B36] Kremers S Reubsaet A Martens M Gerards S Jonkers R Candel M Systematic prevention of overweight and obesity in adults: a qualitative and quantitative literature analysis Obes Rev 2009 19538441 10.1111/j.1467-789X.2009.00598.x

[B37] Pratt CA (Ed) Introduction and overview of worksite studies Supplement Obesity 2007 1s 7s 10.1038/oby.2007.382 18073336

[B38] Glasgow R Terborg J Stryker L Boles Hollis J Take Heart II: replication of a worksite health promotion trial J Behav Med 1997 20 143 161 10.1023/A:1025578627362 9144037

