# Supplementary material for: Changes in skinfold thickness and waist circumference after 12 and 24 months resulting from the NHF-NRG In Balance-project
Source: Int J Behav Nutr Phys Act. 2010 Apr 7;7:26. doi: 10.1186/1479-5868-7-26 (PMC2858095; doi:10.1186/1479-5868-7-26)
Supplement: Additional file 1 — Table S1 - Estimates of treatment effect (B), intercept variance and the intra-class correlation coefficients. Note: Random intercept at worksite and person level, adjusted for baseline age, gender, BMI, education, marital status and smoking status. P-values for differences between intervention and control groups. The ICC (worksite) is the random intercept variance at worksite level divided by the total variance of the outcome measure, the ICC (person) is the random intercept variance at worksite level plus the random intercept variance at person level divided by the total variance of the outcome measure. Abbreviations: sample size (N); standard deviation (SD); unstandardized regression coefficient (B), Confidence Intervals (C.I), estimates of random intercept variance (s2), Standard Error (SE), Intraclass Coefficient (ICC). [file 1479-5868-7-26-S1.DOC]

|  |  | **Intervention** | | **Control** | | |  |  | |  |  |  | |  |  | |
| --- | --- | --- | --- | --- | --- | --- | --- | --- | --- | --- | --- | --- | --- | --- | --- | --- |
|  | **Variable** | **N** | **Mean (SD)** | **N** | | **Mean (SD)** | **B** | **P** | | **95% C.I.** | **Cohen’s *d*** | **s^2^ (SE)** | | **ICC** | | |
|  | Skinfolds thickness (mm) |  |  |  | |  |  |  | |  |  | **Worksite** | **Person** | **Worksite** | | **Person** |
| 12 months | | 294 | -7.4 (11.5) | 164 | -4.7 (9.1) | | -2.52 | | 0.018 | -4.58, -0.45 | 0.26 | 3.010 (2.782) | 102.637 (8.203) | 0.019 | | 0.654 |
| 24 months | | 255 | -10.0 (12.6) | 145 | -5.0 (10.4) | | -4.83 | | <.001 | -6.98, -2.67 | 0.44 |  | |  |
| Waist, cm | |  |  |  |  | |  | |  |  |  |  |  |  | |  |
| 12 months | | 295 | -1.2 (4.9) | 164 | 0.3 (3.3) | | -1.5 | | <.001 | -2.35, -0.65 | 0.37 | 1.667 (1.014) | 19.587 (1.530) | 0.054 | | 0.688 |
| 24 months | | 256 | -0.5 (5.6) | 145 | 1.0 (3.4) | | -1.3 | | 0.005 | -2.18, -0.42 | 0.33 |  | |  |
| Weight, kg | |  |  |  |  | |  | |  |  |  |  |  |  | |  |
| 12 months | | 295 | -0.64 (3.32) | 165 | -0.13 (2.29) | | -0.43 | | 0.2 | -1.08, 0.22 | 0.18 | 0.559 (0.645) | 34.095 (2.351) | 0.014 | | 0.864 |
| 24 months | | 256 | -0.29 (4.25) | 146 | 0.08 (3.16) | | -0.17 | | 0.625 | -0.84, 0.51 | 0.10 |  | |  |
| BMI, kg/m^2^ | |  |  |  |  | |  | |  |  |  |  |  |  | |  |
| 12 months | | 294 | -0.23 (1.2) | 164 | -0.04 (0.8) | | -0.14 | | 0.153 | -0.35, 0.08 | 0.19 | 0.621 (0.389) | 9.311 (0.617) | 0.059 | | 0.942 |
| 24 months | | 255 | -0.11 (1.4) | 145 | 0.03 (1.0) | | -0.05 | | 0.639 | -0.05, 0.23 | 0.12 |  | |  |
